# Detection of changes in regional colonic fermentation in response to supplementing a low FODMAP diet with dietary fibres by hydrogen concentrations, but not by luminal pH

**DOI:** 10.1111/apt.17629

**Published:** 2023-06-30

**Authors:** Daniel So, Chu K. Yao, Paul A. Gill, Phoebe A. Thwaites, Zaid S. Ardalan, Chris S. McSweeney, Stuart E. Denman, Adam F. Chrimes, Jane G. Muir, Kyle J. Berean, Kourosh Kalantar‐Zadeh, Peter R. Gibson

**Affiliations:** ^1^ Department of Gastroenterology Central Clinical School, Monash University and Alfred Health Melbourne Australia; ^2^ Agriculture and Food Commonwealth Scientific and Industrial Research Organisation St. Lucia Australia; ^3^ Atmo Biosciences Melbourne Australia; ^4^ School of Engineering, RMIT University Melbourne Australia; ^5^ School of Chemical Engineering, University of New South Wales Sydney Australia; ^6^ Faculty of Engineering School of Chemical and Biomolecular Engineering, The University of Sydney Sydney Australia

**Keywords:** dietary fibre, fermentation, irritable bowel syndrome, microbiota, resistant starch

## Abstract

**Background:**

Carbohydrate fermentation plays a pivotal role in maintaining colonic health with excessive proximal and deficient distal fermentation being detrimental.

**Aims:**

To utilise telemetric gas‐ and pH‐sensing capsule technologies for defining patterns of regional fermentation following dietary manipulations, alongside conventional techniques of measuring fermentation.

**Methods:**

In a double‐blind crossover trial, 20 patients with irritable bowel syndrome were fed low FODMAP diets that included no extra fibre (total fibre content 24 g/day), or additional poorly fermented fibre, alone (33 g/day) or with fermentable fibre (45 g/day) for 2 weeks. Plasma and faecal biochemistry, luminal profiles defined by tandem gas‐ and pH‐sensing capsules, and faecal microbiota were assessed.

**Results:**

Plasma short‐chain fatty acid (SCFA) concentrations (μmol/L) were median (IQR) 121 (100–222) with fibre combination compared with 66 (44–120) with poorly fermented fibre alone (*p* = 0.028) and 74 (55–125) control (*p* = 0.069), but no differences in faecal content were observed. Luminal hydrogen concentrations (%), but not pH, were higher in distal colon (mean 4.9 [95% CI: 2.2–7.5]) with fibre combination compared with 1.8 (0.8–2.8) with poorly fermented fibre alone (*p* = 0.003) and 1.9 (0.7–3.1) control (*p* = 0.003). Relative abundances of saccharolytic fermentative bacteria were generally higher in association with supplementation with the fibre combination.

**Conclusions:**

A modest increase in fermentable plus poorly fermented fibres had minor effects on faecal measures of fermentation, despite increases in plasma SCFA and abundance of fermentative bacteria, but the gas‐sensing capsule, not pH‐sensing capsule, detected the anticipated propagation of fermentation distally in the colon. The gas‐sensing capsule technology provides unique insights into localisation of colonic fermentation.

**Trial registration:** ACTRN12619000691145.

## Graphical abstract

Changes in regional colonic fermentation in response to supplementing low FODMAP diets with dietary fibres were detected by measurement of luminal hydrogen but not luminal pH.

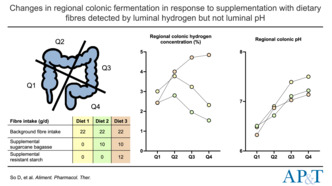

## 1 BACKGROUND

Changes in dietary intake can readily and substantively modify the activity of colonic microbiota by altering the amount and type of substrates available for fermentation. For example, saccharolytic fermentation is associated with putative health benefits, generating gases (carbon dioxide, hydrogen and methane) and short‐chain fatty acids (SCFA) while acidifying the lumen.^1^ Short‐chain fatty acids have a plethora of cellular effects via multiple pathways as shown in vitro and in vivo.^2^ Butyrate, for example, plays essential nutritive roles for the colonic epithelium, exerts concentration‐dependent anti‐inflammatory, differentiative and anti‐tumorigenic effects, but can be toxic at higher concentrations.^2^ Importantly, butyrate mediates its colonic effects topically rather than via systemic delivery following its absorption.^2^, ^3^ Hence, local luminal concentrations and production are of crucial importance to its actions.

Dietary fibres are the major substrate for colonic SCFA production. Accumulated knowledge regarding the fermentation and interaction of specific dietary fibres in the colon has enabled dynamic models of regional fermentation to be developed based upon animal studies^4^, ^5^ and human observations.^6^ These models are founded on three key concepts. First, slowly absorbed or non‐digestible short‐chain carbohydrates, fermentable oligosaccharides, disaccharides, monosaccharides and polyols (FODMAPs), when consumed in usual amounts, are rapidly fermented in the proximal colon leading to their depletion in the distal colon. Excessive fermentation proximally in the colon is potentially injurious, inducing barrier dysfunction and mucosal inflammation,^7^ and may induce symptoms such as abdominal pain and bloating, especially in patients with irritable bowel syndrome (IBS). Reducing dietary intake of FODMAPs ameliorates such symptoms,^7^ but such a strategy potentially reduces fibre intake,^8^ which may reduce delivery of fermentative substrate to the distal colon.^3^

Second, degradation of less rapidly fermented fibres, such as resistant starch (RS), is likely occur over a longer length of the colon but does not spread to the whole colon, unless very large doses are used.^9^ This effect is likely to be due to the sheer amount of fibre available for fermentation. Such a strategy to spread fermentation to the distal colon is not favoured in clinical practice since it is associated with high levels of fermentation in the proximal colon and may induce gastrointestinal symptoms, particularly in patients with IBS.

The third concept involves spreading fermentation of fermentable substrates towards the distal colon, but without excessive fermentation proximally. This has been achieved by adding a non‐ or poorly fermentable fibre with fermentable fibres. Initial experiments were performed in rats in which luminal concentrations of fermentation products, specifically SCFA, were higher in the distal colon by combining wheat bran, a bulking fibre, with resistant starch than could be achieved by either alone.^5^ The potential health value of such an approach was supported by suppression of tumorigenesis in rats using the fibre combination strategy,^5^ likely to be related to increased delivery of butyrate to the distal epithelium.^10^ Given the methodological difficulties in proving this directly in humans, a study was performed in pigs, whose colonic function better mimics that of humans. Indeed, a combination of RS and wheat bran spread fermentation evenly around the colon.^4^ The increase in substrate and fermentation in the distal colon using this fibre combination was subsequently shown in healthy humans using large doses of RS and measuring SCFA concentrations in the faeces where residual starch was increased in the faces in association with wheat bran and RS supplementation.^9^, ^11^ In a recent study that utilised magnetic resonance imaging of the colon, the effect of psyllium (poorly fermentable) and inulin (fermentable) showed the spread of fermentation to the distal colon in humans.^12^ Two potential mechanisms by which the effect of a poorly fermented fibre can lead to a distal spread of fermentation have been identified. The gel‐structures formed by viscous fibres reduce the accessibility of fermentable fibres to the microbiota, which slows fermentation in vivo, but does reduce the amount of in vitro.^12^ The other potential contributing factor is that the transit‐hastening effects of bulking fibres propel the fermentable substrate along the colon.^4^, ^6^

Measurement of regional colonic fermentation is problematic. Conventional techniques of assessing the delivery of SCFA to the colonic mucosa in humans comprise measurement of metabolite concentrations in faeces, which are more likely to be representative of fermentation in the distal colon and rectum and poorly represent their production,^13^, ^14^ and are subject to the artefacts of ongoing fermentation of residual carbohydrates in the faeces ex vivo.^15^ Evaluation of breath hydrogen and methane, and plasma SCFA concentrations, can provide indirect insights into overall fermentation occurring in the intestine, but gives limited information on where the fermentation is occurring, and also has limited reproducibility.^2^ Similarly, taxonomic and metabolic analyses of faecal microbiota offer limited insights into local processes.

Telemetric capsule technologies offer the opportunity to overcome such limitations via localised, real‐time assessments of intraluminal metabolite concentrations in the ambulant person without physiological disruption. Luminal pH, measured by the wireless motility capsule (WMC), has been applied as marker of luminal fermentation due to the acidification related to SCFA, succinate and lactate formation, but pH is the sum of metabolic activities and hence not specific to fermentation.^16^ In contrast, luminal hydrogen concentration offers a highly specific measure of local fermentation since hydrogen is only produced via saccharolytic fermentation and is rapidly consumed via microbial metabolic pathways or via absorption to systemic circulation.^1^ The development of a telemetric capsule that samples volatile molecules, including simple gases such as hydrogen, through a semipermeable membrane offers a more specific option.^17^

Thus, the current study aimed first, to evaluate the hypothesis that the WMC and gas‐sensing capsule can detect changes in colonic fermentation and its distribution induced by manipulation of the types of dietary fibres consumed in humans. Second, the ability of luminal pH and hydrogen concentrations to detect changes in regional fermentation was compared. Third, the study aimed to compare the telemetric findings with those using conventional techniques of assessing fermentation. To do this, patients with IBS were studied under carefully controlled conditions by feeding them diets restricted in FODMAP content (to reduce proximal fermentation) and supplemented or not with sugarcane bagasse, a poorly fermented fibre comprised of fractions highly resistant to fermentation (~50% cellulose, 25%–35% hemicelluloses, 15%–25% lignin)^18^, ^19^ and possessing stool bulking properties,^20^ alone or with a moderately fermentable RS (to change regional fermentative profiles). Regional changes in the colonic lumen were measured via tandem ingestion of telemetric capsules.

## 2 METHODS

### 2.1 Participants

The participants have been previously described in detail.^20^ Briefly, symptomatic subjects with IBS as defined by Rome IV criteria^21^ were recruited. Exclusion criteria comprised gastrointestinal or metabolic comorbidities, previous abdominal surgery, currently following a therapeutic diet (e.g. low FODMAP), and use of antibiotics, prebiotics and/or probiotics in the previous 4 weeks. All participants provided written informed consent. The trial was registered with the Australian New Zealand Clinical Trials Registry (ACTRN12619000691145) with ethical approval provided by the Monash University Human Research Ethics Committee (Reference: 12804). All authors approved the final version of the manuscript.

### 2.2 Trial design and procedures

The trial design and most procedures have been previously described in detail.^20^ Briefly, participants maintained typical dietary habits during a 7‐day baseline before being randomised to receive one of three 14‐day dietary interventions (detailed below). Both participants and investigators were blinded to the diets. The three dietary interventions were separated by a ≥21‐day washout, where participants resumed typical dietary habits.

Trial procedures are illustrated in Figure S1. Assessments made via telemetric capsules, on faecal and plasma metabolite concentrations, as well as on faecal microbiota, are presented in the current report. Briefly, during the 7‐day baseline, participants collected all faeces passed on days 3–7 in individual plastic containers, which were immediately sealed and placed at −20°C in a provided portable freezer. On day 7, participants collected an independent microbiota sample using a specialised kit (OMNIgene.GUT OM‐200, DNA Genotek). During each 14‐day dietary intervention, participants presented to the trial centre to provide a blood sample after fasting overnight on day 9, with a sub‐group agreeing to ingest two telemetric capsules (detailed below) in tandem as an optional assessment. Faecal and microbiota samples were collected during days 10–14 and day 14 during each dietary intervention respectively.

### 2.3 Interventional diets

The dietary interventions (designated ‘Control’, ‘Sugarcane’, ‘Combination’) were delivered via controlled feeding, where most food was provided to participants, as previously described.^20^ Briefly, the Control diet comprised a base low FODMAP diet; the Sugarcane diet comprised the base low FODMAP diet supplemented with 10 g/day fibre from sugarcane bagasse (Tamu Pty. Ltd.); the Combination diet comprised the base low FODMAP diet supplemented with 10 g/day fibre from sugarcane bagasse and 12 g/day RS from high‐amylose starch (Hi‐Maize 1043, Ingredion). Other than fibre content, the diets were nutritionally identical (Table S1).

### 2.4 Telemetric assessments

The telemetric capsules studied were the WMC (SmartPill™, Medtronic Australasia) that measures luminal pH, pressure and temperature and gas‐sensing capsule (Atmo Gas Capsule, Atmo Biosciences) that detects a range of gas concentrations, predominantly hydrogen, carbon dioxide and oxygen, and temperature (Figure S2). On day 9 during each dietary intervention, a sub‐group of participants ingested the capsules in tandem in a random order after consuming breakfast comprising cereal with 250 mL lactose‐free milk (Figure S1), before fasting for 4 h and resuming the intervention diet as previously outlined in detail.^20^ The 4‐h fasting period was associated with delayed gastric emptying in some patients,^20^ but this did not change the ability to assess colonic gas patterns. Participants wore receivers corresponding to each capsule until their passing, confirmed by fall in temperature, signal loss following bowel movement and/or visual confirmation in collected faecal samples.

Colonic transit time was calculated from the time each capsule reached the ileocaecal junction to its excretion, as previously reported and validated.^22^, ^23^ Colonic pH was examined as an average across the entire colon, together with nadir and peak pH and their timing after the ileocaecal valve. Colonic hydrogen concentrations were expressed as a percentage of the gas detected within the lumen, together with peak concentration and its timing after the ileocaecal valve. The colon was segmented into quartiles based on relative transit time^24^ to enable regional fermentation to be assessed.

### 2.5 Faecal metabolite assessments

Faecal samples were pooled, homogenised and analysed in triplicate as previously described.^20^, ^25^ Briefly, thawed stool samples were spiked with three times the volume of internal standard (1.68 mM heptanoic acid); homogenised and centrifuged (2000 ***g***, 10 min, 4°C) with 300 μL of supernatant added to 0.2 μm filter vials containing 10 μL 1 M phosphoric acid. The vials were analysed for SCFA and branched‐chain fatty acids (BCFA) concentrations using an Agilent GC6890 (Agilent Technologies Australia; Australia) coupled to a flame‐ionisation detector. Analyses were conducted in triplicate. A coefficient of variation <15% was taken as a valid result. Faecal pH was measured with a calibrated pH probe (Five‐Go pH meter, Mettler‐Toledo) with the sample warmed to 25°C.

### 2.6 Plasma metabolite assessments

Plasma samples were analysed in duplicate for SCFA content as previously described.^25^ Briefly, 300 μL of plasma was spiked with 200 μM internal standard (1.68 mM heptanoic acid) and acidified using 10% sulfosalicylic acid, with 3 mL diethyl ether solvent added. The mixture was vortexed and then centrifuged (400 ***g***, 2 min, 4°C) to clarify the organic layer, which was transferred into 50 μL 0.2 M sodium hydroxide. The SCFA‐containing alkaline solution was concentrated by evaporation over nitrogen. The pellet produced was dissolved in 30 μL 1 M phosphoric acid, transferred into cold vials and analysed using an Agilent GC6890 as described above. Concentrations for acetate, propionate and butyrate were determined by the average of the duplicate results. A coefficient of variation <20% was taken as a valid result.

### 2.7 Microbiota assessments

DNA extractions were carried out using the cetyltrimethylammonium bromide (CTAB) method of Brookman and Nicholson^26^ with modifications as follows: 200 mg faecal sample was homogenised with 200 mg of silica–zirconium beads (1:1 mixture of 0.1 and 1.0 mm beads [Biospec]) and 800 mL of CTAB buffer in a Fastprep‐24 high‐speed benchtop homogeniser (MP Biomedicals) on maximum speed for 2 min, twice. Samples were incubated at 70°C for 20 min and centrifuged at 10,000 ***g*** for 10 min, and the supernatant was mixed with an equal volume of 25:24:1 phenol–chloroform–isoamyl alcohol, followed by DNA precipitation with isoamyl alcohol. The yield and purity of extracted DNA were assessed with a NanoDrop 8000 spectrophotometer (Thermo Fisher Scientific).

Bacterial populations were characterised by amplifying the v3‐v4 region of the 16S rRNA gene using established primers (F341/R806).^27^, ^28^ Each DNA sample was amplified using target‐specific primers and a unique barcode combination as described previously.^29^ Amplification products were visualised by gel electrophoresis. Product quantities were calculated, and an equal molar amount of each target product was pooled. The pooled target products were run in a 1.5% agarose gel; bands were visualised and excised under blue light trans‐illumination. The amplicons were gel purified with a QIAquick Gel Extraction Kit (Qiagen) prior to submission for 2 × 250 bp Illumina MiSeq sequencing.

Paired‐end short‐read sequence data generated on the Illumina MiSeq was processed using the VSEARCH package.^30^ De‐multiplexed paired‐end sequences were passed through cutadapt for primer removal^31^ and then merged prior to sequence quality filtering, followed by error correction,^32^ chimera checking,^33^ and clustering of sequences to Amplicon sequence variants (ASVs).^34^ Taxonomic classification of bacterial ASVs was done using the IDTAXA algorithm implemented in the DECIPHER R package against the SILVA SSU r132 training set.^35^

### 2.8 Statistical analysis

Sample size estimations were calculated on faecal output as published.^20^ Data presented in this report were analysed per‐protocol with comparisons made across the three dietary interventions using R statistical software and GraphPad Prism. Analyses were performed using linear mixed‐effects modelling fit by restricted maximum likelihood using the lme4 package. The intervention periods were modelled as a fixed effect, with participants and diet order modelled as random effects. Where model residuals were not normally distributed, data were normalised by log‐transformation for analyses, but presented as non‐transformed values. Multiple comparisons between the three diets were made using the multcomp package with no post hoc corrections made. Differences were considered significant where *p* ≤ 0.05.

Identification of ASVs contributing to a microbiome signature characterising each dietary intervention was performed in R using the mixOmics package.^36^ A supervised sparse Partial Least Squares Discriminant Analysis (sPLS‐DA) was used with centred log ratio transformation of the count data and a repeated measurement design using the patient ID to account for the high inter‐subject variation, characteristic of microbiome data. The number of selected ASV's for each component and number of components to use in the final model was optimised and validated using cross‐validation (10‐fold), repeated 100 times. The lowest prediction error rate for each cross‐validation was then used to determine the selected ASV features at each component and used as parameters in the final model. Sample plots displaying similarities between samples in a reduced space spanning the first three latent components of the final sPLS‐DA model were produced using the calculated component scores. Confidence ellipse on the sample plots is for each diet, reflecting the 95% confidence level for a pairwise confidence region.

## 3 RESULTS

### 3.1 Participants successfully adhered to dietary interventions

Twenty participants, 19 female, median age 34 (range: 19–61) years, body mass index 23 (range: 16–31) kg/m^2^, completed the trial. Eighteen consented for capsule investigations. The flow of participants and successful capsule investigations are shown in Figures S3 and S4. As previously reported, dietary adherence was excellent: participants consumed ≥89% of the fibre‐containing meals across the diets, with minimal deviations from the diets reported.^20^ The participants successfully reduced consumption of FODMAPs during all interventions and intake of fibre and/or RS was increased as planned during the Sugarcane and Combination diets.^20^

### 3.2 Effect of diets on plasma SCFA and faecal metabolite concentrations

**FIGURE 1:**
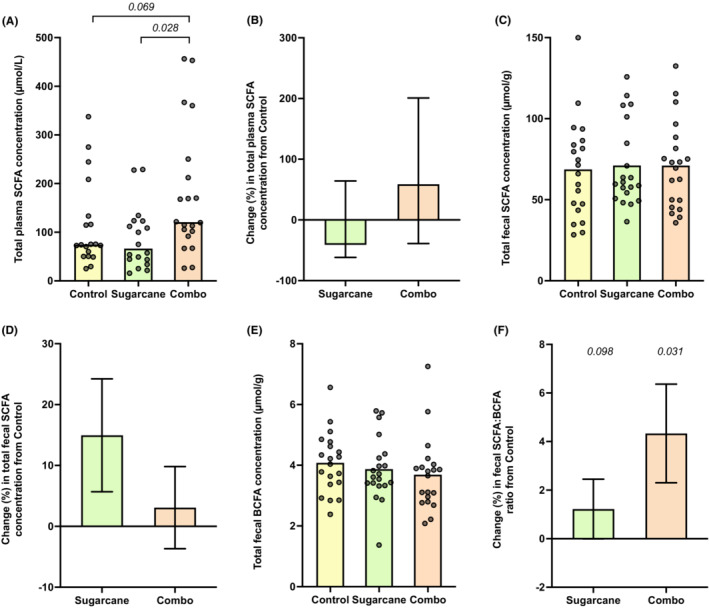
Metabolite concentrations in plasma and faeces across the dietary interventions. (A) Total plasma short‐chain fatty acid (SCFA) concentration (bar as median). (B) Relative change in total plasma SCFA concentration relative to Control (mean ± SEM). (C) Total faecal SCFA concentration (bar as mean). (D) Relative change in total faecal SCFA concentration relative to Control (mean ± SEM). (E) Total faecal branched‐chain fatty acid (BCFA) concentration (bar as mean). (F) Relative change in faecal SCFA:BCFA relative to Control (mean ± SEM).

Total plasma SCFA concentrations were median 64% and 83% higher in the Combination diet compared with those during the Control (*p* = 0.069) or Sugarcane diets (*p* = 0.028) respectively (Table 1; Figure 1). Across the diets, 92%–95% of plasma SCFA was acetate. Propionate and butyrate plasma concentrations were near or below the lower limit of the assay and are not presented.

**TABLE 1:** Faecal and plasma metabolite concentrations across the dietary intervention periods, including concentrations of short‐chain fatty acids (SCFA), branched‐chain fatty acids (BCFA) and faecal pH.

	Control	Sugarcane	Combination
Faecal metabolite concentrations (μmol/g)			
Total SCFA	68.7 (46.4–84.5)	60.2 (53.4–88.9)	71.3 (48.6–83.4)
Acetate	42.9 (28.1–51.4)	37.4 (32.4–55.7)	42.2 (31.8–55.2)
Propionate	11.6 (6.2–16.0)	11.3 (7.8–17.9)	10.3 (7.3–14.3)
Butyrate	10.1 (7.3–15.8)	10.3 (8.5–13.2)	10.6 (7.8–14.0)
Total BCFA	4.1 (3.4–4.7)	3.7 (3.4–4.3)	3.7 (2.9–4.0)
SCFA:BCFA ratio	15.3 (11.8–19.7)	16.7 (13.8–19.9)	19.8 (11.6–31.5)
pH	6.6 (6.4–6.8)^a, b^	6.8 (6.6–6.9)^a^	6.7 (6.5–6.9)^b^
Plasma metabolite concentrations (μmol/L)			
Total SCFA	74.0 (55.3–124.7)	66.2 (43.9–120.2)^a^	121.0 (99.5–221.8)^a^
Acetate	72.9 (54.4–115.9)	64.5 (43.0–118.3)^a^	113.0 (91.8–193.3)^a^

*Note*: Data shown as median (IQR) and analysed via linear mixed models. Significant differences (*p* ≤ 0.05) between the dietary interventions shown via shared superscripts.

Total faecal concentrations of SCFA were similar across the diets (Figure 1), as were the major SCFA (Table 1) and their relative proportions (data not shown). While no significant differences in the concentrations of BCFA were observed across the diets, SCFA:BCFA ratios were higher in the Combination compared with the Control diet (*p* = 0.031). The pH of pooled faecal samples was higher during the Sugarcane compared with Control (median difference 0.14, *p* = 0.002) and Combination diets (0.11, *p* = 0.049).

### 3.3 Colonic pH profiles from the wireless motility capsule

**FIGURE 2:**
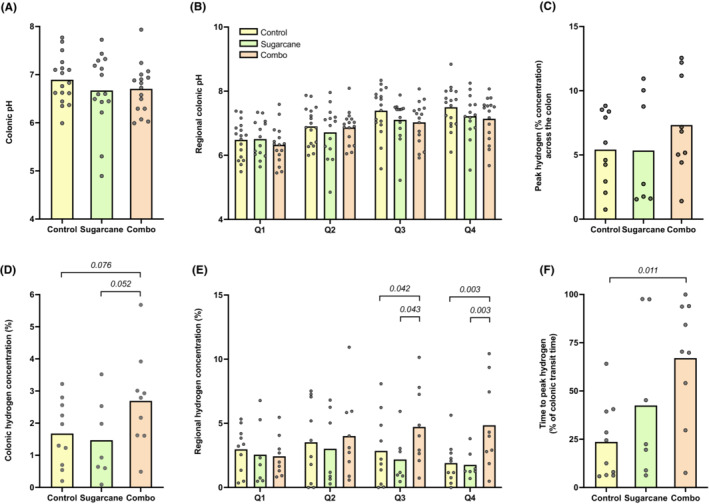
Luminal pH and hydrogen profiles across the dietary interventions. (A) Overall colonic pH (bar as mean). (B) Regional colonic pH per quartile of relative colonic transit time (bar as mean). (C) Overall colonic hydrogen concentration (% of gas detected within the lumen; bar as mean). (D) Peak hydrogen concentration (bar as mean). (E) Regional colonic hydrogen concentration per quartile of relative colonic transit time (bar as mean). (F) Time to peak hydrogen concentration relative to colonic transit time (bar as mean).

**FIGURE 3:**
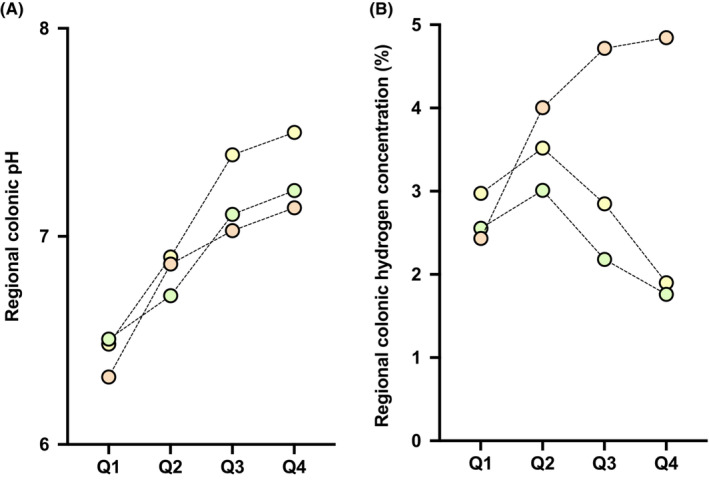
(A) Luminal pH and (B) hydrogen profiles per quartile of relative colonic transit time. Mean pH or hydrogen concentration (% of gas detected within the lumen;) per quartile shown.

No differences in overall colonic pH or the level and timing of the pH nadir were observed across the diets (Table 2; Figure 2). Peak pH was higher during the Control compared with the Sugarcane (mean difference 0.4; *p* = 0.035) and Combination diets (0.5; *p* = 0.012), occurring numerically later in the Combination diet (at median 80% of colonic transit time) compared with that in the Control (67%, *p* = 0.423) and Sugarcane diets (57%, *p* = 0.092). Proximal‐to‐distal gradient of colonic pH increased similarly across the three diets with no statistical differences (Figure 3).

**TABLE 2:** Colonic pH profiles across the dietary intervention periods, including overall and regional colonic pH, as well as pH nadir and peak metrics.

	Control	Sugarcane	Combination
pH			
Overall	6.9 (6.6–7.2)	6.7 (6.2–7.1)	6.7 (6.4–7.0)
Quartile 1	6.5 (6.2–6.8)	6.5 (6.2–6.8)	6.3 (6.0–6.6)
Quartile 2	6.9 (6.6–7.2)	6.7 (6.2–7.2)	6.9 (6.6–7.1)
Quartile 3	7.4 (7.0–7.8)	7.1 (6.7–7.5)	7.0 (6.7–7.4)
Quartile 4	7.5 (7.2–7.8)	7.2 (6.8–7.6)	7.1 (6.8–7.5)
pH nadir and peak metrics			
Nadir	5.4 (5.4–5.7)	5.7 (5.4–6.0)	5.4 (5.2–5.7)
Time to nadir (from ileocaecal valve; hours)	1.5 (0.3–2.7)	1.3 (0.9–2.1)	1.7 (0.7–2.1)
Time to nadir (% of colonic transit time)	4.1 (2.3–14.4)	5.5 (3.8–13.3)	4.7 (3.6–14.9)
Peak	8.4 (8.2–8.6)^a, b^	8.0 (7.7–8.4)^a^	7.9 (7.7–8.2)^b^
Time to peak (from ileocaecal valve; hours)	24.1 (8.8–34.9)	11.1 (4.0–30.9)	13.4 (8.8–20.5)
Time to peak (% of colonic transit time)	67.2 (51.9–84.0)	57.0 (30.8–97.4)	80.0 (55.6–90.1)

*Note*: Data shown as mean (95% CIs) for pH values, median (IQR) for time to pH nadir and peak, and analysed via linear mixed models. Significant differences (*p* ≤ 0.05) between the dietary interventions shown via shared superscripts.

### 3.4 Colonic hydrogen profiles from the gas‐sensing capsule

Overall colonic hydrogen concentration tended to be higher during the Combination compared with Control (mean difference 1%; *p* = 0.076) and Sugarcane diets (1%; *p* = 0.052) (Table 3; Figure 2). Peak hydrogen concentration was similar across the diets but occurred later during the Combination compared with Control diet; when expressed in terms of absolute time from ileocaecal junction, the median difference was 7 h (*p* = 0.071) and, relative to colonic transit time, the median difference was 52% (*p* = 0.011). Regionally, hydrogen concentration tended to exhibit a proximal‐distal fall during the Control and Sugarcane diets, and appeared to increase across proximal‐to‐distal quartiles in association with the Combination diet. In Quartile 4, hydrogen concentration was more than two‐fold higher during the Combination diet compared with the Control (*p* = 0.003) and Sugarcane diets (*p* = 0.003) (Figure 3).

**TABLE 3:** Colonic hydrogen profiles across the dietary intervention periods, including overall and regional hydrogen concentrations (% of gas detected in the lumen), and peak hydrogen concentration metrics.

	Control	Sugarcane	Combination
Hydrogen concentration (% of gas detected within the lumen)			
Overall	1.7 (0.9–2.4)	1.5 (0.3–2.6)	2.7 (1.5–3.9)
Quartile 1	3.0 (1.1–4.2)	2.6 (0.2–4.9)	2.4 (1.2–3.6)
Quartile 2	3.5 (1.3–5.7)	3.0 (0.3–5.7)	4.0 (1.5–6.5)
Quartile 3	2.9 (0.9–4.8)^a^	2.2 (0.4–4.0)^b^	4.7 (2.3–7.1)^a, b^
Quartile 4	1.9 (0.7–3.1)^a^	1.8 (0.8–2.8)^b^	4.9 (2.2–7.5)^a, b^
Hydrogen peak metrics			
Peak concentration (%)	5.4 (3.3–7.5)	5.3 (1.3–9.4)	7.3 (4.4–10.3)
Time to peak (from ileocaecal valve; hours)	4.2 (2.8–7.2)	6.5 (3.5–9.1)	11.4 (6.7–32.1)
Time to peak (% of colonic transit time)	18.5 (11.4–36.7)^a^	22.4 (14.2–71.4)	70.4 (54.1–93.7)^a^

*Note*: Data shown as mean (95% CIs) for hydrogen concentrations, median (IQR) for time to peak hydrogen, and analysed via linear mixed models. Significant differences (*p* ≤ 0.05) between the dietary interventions shown via shared superscripts.

### 3.5 Faecal microbiota composition

**FIGURE 4:**
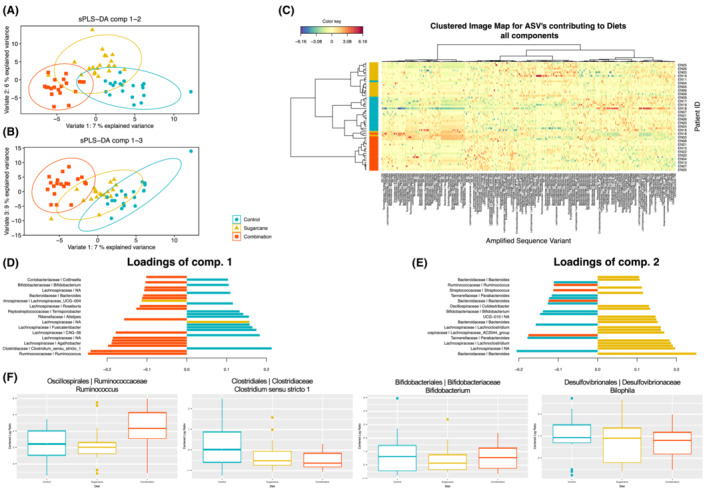
Influence of three interventional diets on faecal microbiota via 16S amplicon sequencing. (A, B) Sample analysis plots for sparse partial least‐squares discriminant analysis (sPLS‐DA) projected into the first three components. (C) Clustered heatmap after sPLSDA modelling for the optimal selected features with the cell colours depicting the centred log ratio (CLR)‐transformed abundance values. (D, E) The loading values (importance) of the top 30 contributing features for the first and second components. (F) Differences in relative abundance (CLR transformed) of amplified sequence variants (ASVs), with selected boxplots on the basis of marked expansion of *Ruminococcus* when exposed to the sugarcane bagasse and resistant starch, but no differences in other relevant ASVs (*Bifidobacterium* and *Bilophila*). Bar lengths correspond to the importance (the multivariate regression coefficient for that particular feature on each component) of the feature in the final sPLS‐DA model (A, B).

The compositions of faecal microbiota were compared across the three diets (Figure 4). There were no differences in alpha‐diversity or richness (data not shown). On unsupervised principal component analysis (PCA), there were no overall differences in composition. However, when the most discriminative features in the data were analysed by sparse partial least‐squares discriminant analysis, clear differentiation in microbial composition between the Combination and Control diets with less discrimination between the Sugarcane and the other two diets was observed, as shown in the PCA plots and heatmap. For individual ASVs, the major differences in general were a marked increase in relative abundance of *Ruminococcus* and reduction in *Clostridium* sensu stricto 1 in the Combination diet. One highly abundant *Faecalibacterium* ASV was positively associated with the Combination diet, but overall this genus was not different between treatments. No differences were observed in the density of Bifidobacteria or *Bilophila*.

## 4 DISCUSSION

Given the pivotal position of bacterial fermentation in the colon for the structure of the microbiota, and for the health of the mucosa and the organism in general, it is alarming that our ability to evaluate fermentation regionally in the large bowel is so poor. It has relied upon extrapolation from studies in animals with very different intestinal physiology, upon measurement of faecal indices like SCFA concentrations that have poor correlation with activities more proximally, and more recently, directly using magnetic resonance imaging (MRI).^12^ The current study addressing capsule technologies with telemetric transmission of data has shown the limited worth of faecal measurements and luminal pH, and the unique value of hydrogen concentrations to reflect fermentative activities regionally in ambulatory humans under physiological conditions.

In designing the dietary manipulation of this study, four key factors were sought. First, the dietary intake had to be strictly controlled such that unevenness of background intake would not introduce confounding effects. This was achieved by providing nearly all food and carefully monitoring intake. The background diets were very similar across the three arms. Second, the quantum of fibre supplementation had to be within the scope of normal clinical practice and supra‐physiological doses avoided.^37^ Patients with IBS were studied since FODMAP and fibre intake is of direct relevance to them, with doses used modest and well‐tolerated.^38^ Third, that fermentation patterns would be altered in its total amount and distribution along the colon was important so that shifts in regional fermentation might be detected. To do this, a fermentable substrate (RS) was used together with a poorly fermented fibre (sugarcane bagasse) since such a combination of fibres with these characteristics has been demonstrated by studies of healthy subjects and patients with IBS.^6^, ^11^, ^12^ The background diet was low in FODMAP content as this would specifically reduce proximal fermentation, as shown via breath gas.^39^ That the supplemented fibres differed markedly in fermentability had already been shown in vitro.^19^ Hence, it was anticipated that distal fermentation would be enhanced by the sugarcane bagasse/RS combination. Finally, a cross‐over design was essential to permit the evaluation of changes in indices that have considerable inter‐subject variance, such as SCFA concentrations and faecal microbiota.

That overall colonic fermentation was increased by the combination fibre supplementation was supported by increases in plasma SCFA concentrations in association with the Combination diet, despite low systemic bioavailability due to their metabolism by the epithelium and the liver^2^ and by increases in relative abundances of taxa capable of saccharolytic fermentation, especially *Ruminococcus* and one species of *Faecalibacterium*.^38^ Limitations of conventional approaches to the measurement of colonic fermentation in humans were highlighted by the lack of differences in concentrations of fermentative metabolites, particularly SCFA, in the faeces. This is likely to be due to their rapid absorption following production, supported by observations that their concentration was more a reflection of transit time than fibre intake.^40^ There was, however, one indicator of enhanced carbohydrate fermentation in the distal colon by the increased SCFA:BCFA ratio reflecting a relative increase of carbohydrate over protein fermentation that has been well documented in studies of faecal slurries ex vivo.^41^ Both telemetric capsules appear to offer limited utility for the measurement of overall fermentation. Indeed, while colonic pH was numerically lower and hydrogen concentration higher with the Combination diet, these were not statistically significant when compared with the paired results associated with a lower fermentable load. Reasons for the lack of differences are that net pH is affected by the contribution of other metabolites, such as ammonia, a product of protein fermentation, which are weak bases,^42^ and overall hydrogen concentrations represent the net of production and disposal, the efficiency of which is likely to change with changing structure and capabilities of microbial populations across timepoints in an individual. Hence, faecal, plasma and luminal measures are not reliable markers of total colonic fermentation.

However, the location of fermentation within the colon is as important as its magnitude. As outlined above, clinical problems may arise from too much in the proximal colon (symptom genesis and mucosal injury) or too little in the distal colon (loss of protection from carcinogenesis or impairment of barrier function).^7^ Conventional faecal and plasma measures provide few insights, but telemetric measurement of metabolites that are rapidly depleted by either metabolism or absorption at the site of production provides a unique opportunity to define variations along the colon. While localisation of key landmarks enabling assessment of regional gastrointestinal transit has been validated for the WMC and gas‐sensing capsule,^23^ the localisation within the large bowel itself is less precise and depends upon the net movement of the capsules from proximal to distal colon. Hence, information from the colon was divided into quartiles with the first and last clearly being associated with proximal and distal colonic events, respectively, and the time to peak concentrations or nadir of the pH were applied in order to judge quantitative distribution of fermentation.

Luminal pH may be a useful marker for the degree of carbohydrate fermentation in the proximal colon,^3^ but this was not investigated in the current study. The value of pH in the distal colon may be considerably reduced since more metabolites that contribute to the net pH, such as ammonia described earlier, occur there. Indeed, the pattern of pH across the quartiles was similar across all dietary arms and, therefore, provided little insight into changes in distal fermentation patterns due to its non‐specificity.

In contrast, the specificity of hydrogen production to carbohydrate fermentation and the likely expectation that, within one individual, the hydrogen‐disposal mechanisms will be similar along the colon, it might be anticipated that, within a single study, variations of hydrogen concentrations might reflect differences in production. Indeed, the gas‐sensing capsule showed that fermentation can be pushed distally by the Combination diet via the timing of the peak hydrogen concentrations and the pattern of hydrogen concentrations across the quartiles of colon. Thus, fermentation was increased distally for the Combination diet whereas it was diminished distally with the Control and Sugarcane diets, as predicted from the dietary designs. Hence, the gas‐sensing capsule enables changes to the distribution of fermentation in the colon to be identified without the need for animal experiments or complex and very expensive methodology in humans.

Strengths of this study include the robust manner by which the trial was conducted and the clinical applicability of the findings, given the tolerance of dietary interventions within physiological range and clinical responses.^20^ This study provided the first evidence that regional changes in colonic fermentation in the ambulant human can be detected by direct luminal assessments. Furthermore, multiple indices evaluated fermentative responses, with conventional assessments of faecal and plasma metabolites, as well as faecal microbiota composition, combined with telemetric technology, and these were used to interrogate and contextualise the effects.

Limitations of the study mostly related to its pilot nature with its limited statistical power. First, the limited numbers related in part to the smaller number of participants who undertook the capsule investigations and was compounded by the availability of technically satisfactory data. Second, our interpretation of these data is based on the premise that the addition of poorly fermented fibre to fermentable fibre can spread fermentation distally, and that sugarcane bagasse has functional characteristics similar to poorly fermented fibres used previously. While both have previously been demonstrated,^4^, ^20^ an additional arm where RS was supplemented alone would have enabled direct and explicit validation of the model used. Third, use of a higher overall fibre load, more fermentable fibres and/or lower doses in the fibre control arm may have more pronounced effects to improve the power of the study, but generalisability of the findings and application to clinical scenarios may have been compromised, and the potential for higher fibre loads reaching the distal colon without needing to be combined^9^ would be a nuanced question requiring specific investigation. Fourth, while limited, breath hydrogen profiles were not assessed in the study, which may have offered value as another fermentative index for comparison, and enabled ready comparison with other studies. Finally, the development and validation of anatomical landmarks within the colon will enable more accurate assessment of regional fermentation, as the indices utilised in this study may not have captured the nuances of colonic transit, such as propulsive or retrograde movements that occur within the lumen,^43^ or the effects of particularly rapid transit.

In conclusion, concomitant supplementation of poorly fermented fibre with fermentable fibre in patients with IBS initiated on a low FODMAP diet enhanced saccharolytic fermentation while propagating fermentative activities towards the distal colon. The application of telemetric capsule technologies, especially the gas‐sensing capsule, provided insights into localisation of colonic fermentation beyond the capabilities of conventional stool measurements. Not only does this work provide novel perspectives of how different types of fibre are utilised in the colon, but it may also change how colonic fermentation is assessed in moving forward. Future iterations of this technology, capable of measuring gases reflecting hydrogen disposal pathways, such as methane and hydrogen sulphide, together with localisation of the capsule within the colon, may enable the additional aspect of the ‘black box’ of colonic fermentation in humans to be unlocked.

## AUTHOR CONTRIBUTIONS

**Daniel So:** Data curation (lead); formal analysis (lead); investigation (lead); methodology (equal); project administration (lead); visualization (lead); writing – original draft (lead). **Chu K. Yao:** Methodology (equal); supervision (supporting); writing – review and editing (supporting). **Paul Gill:** Investigation (supporting); writing – review and editing (supporting). **Phoebe A. Thwaites:** Investigation (supporting); writing – review and editing (supporting). **Zaid S. Ardalan:** Investigation (supporting); writing – review and editing (supporting). **Chris McSweeney:** Data curation (supporting); formal analysis (supporting); funding acquisition (equal); investigation (supporting); visualization (supporting); writing – review and editing (supporting). **Stuart Denman:** Data curation (supporting); formal analysis (supporting); investigation (supporting); visualization (supporting); writing – review and editing (supporting). **Adam Chrimes:** Data curation (supporting); investigation (supporting); visualization (supporting); writing – review and editing (supporting). **Jane G. Muir:** Conceptualization (equal); funding acquisition (equal); methodology (equal); supervision (lead); writing – review and editing (supporting). **Kyle J. Berean:** Conceptualization (equal); data curation (supporting); funding acquisition (equal); investigation (supporting); visualization (supporting); writing – review and editing (supporting). **Kourosh Kalantar‐zadeh:** Conceptualization (equal); funding acquisition (equal); visualization (supporting); writing – review and editing (supporting). **Peter Gibson:** Conceptualization (equal); funding acquisition (equal); methodology (equal); supervision (supporting); visualization (supporting); writing – review and editing (lead).

## FUNDING INFORMATION

This work was funded in part by the National Health and Medical Research Council of Australia (GNT1154969) and funded in part by Tamu Innovations through the Graduate Research Industry Partnership at Monash University. DS was supported by scholarships from Monash University via the Faculty of Medicine, Nursing and Health Science and the Graduate Research Industry Partnership. JGM was supported by a Research Fellowship from the National Health and Medical Research Council of Australia (APP1136988). Gas‐sensing capsules used were donated by Atmo Biosciences.

## CONFLICT OF INTEREST STATEMENT

DS: Previous employee and shareholder of Atmo Biosciences. CKY: Recipient of research support from Atmo Biosciences. PAG: None. PAT: None. ZSA: None. CSMcS: None. SED: None. AFC: Employee and shareholder of Atmo Biosciences. JGM: None. KJB: Employee and shareholder of Atmo Biosciences. KK–Z: Has consulted for Atmo Biosciences. PRG: Consultant or advisory board member for Anatara, Atmo Biosciences, Immunic Therapeutics, Novoviah, Intrinsic Medicine, Topas and Comvita; recipient of research grants for investigator–driven studies from Atmo Biosciences and Nerva, and speaker honoraria from Dr Falk Pharma; shareholder in Atmo Biosciences. The Department of Gastroenterology, Monash University and Alfred Health, financially benefits from the sales of a digital application, a book and booklets, and online courses on the FODMAP diet.

## AUTHORSHIP

*Guarantor of the article*: Peter R. Gibson.

## Supporting information


Appendix S1.
