# Supplementary material for: Detection of changes in regional colonic fermentation in response to supplementing a low FODMAP diet with dietary fibres by hydrogen concentrations, but not by luminal pH
Source: Aliment Pharmacol Ther. 2023 Jun 30;58(4):417–28. doi: 10.1111/apt.17629 (PMC10946934; doi:10.1111/apt.17629)
Supplement: Supplementary file 1 — Appendix S1. [file APT-58-417-s001.docx]

**SUPPLEMENTARY INFORMATION**

**Detection of changes in regional colonic fermentation in response to supplementing a low-FODMAP diet with dietary fibres by hydrogen concentrations, but not by luminal pH**

Daniel So^1^, Chu K Yao^1^, Paul A Gill^1^, Phoebe A Thwaites^1^, Zaid S Ardalan^1^, Chris S McSweeney^2^, Stuart E Denman^2^, Adam F Chrimes^3,4^, Jane G Muir^1^, Kyle J Berean^3,4^, Kourosh Kalantar-Zadeh^5,6^, Peter R Gibson^1^

**Institutions**

^1^ Department of Gastroenterology, Central Clinical School, Monash University; Melbourne, Australia

^2^ Agriculture and Food, Commonwealth Scientific and Industrial Research Organisation; St. Lucia, Australia

^3^ Atmo Biosciences; Melbourne, Australia

^4^ School of Engineering, RMIT University; Melbourne, Australia

^5^ School of Chemical Engineering, University of New South Wales; Sydney, Australia

^6^ School of Chemical and Biomolecular Engineering, Faculty of Engineering, The University of Sydney; Sydney, Australia

Supplementary Figure 1

Overview of data collection procedures during (**A**) the baseline period and (**B**) each dietary intervention. Assessments highlighted in red were not discussed in this article.

Abbreviations: DASS-21, Depression, Anxiety and Stress Score questionnaire; D-FIS, daily fatigue impact scale questionnaire; IBS-QoL; irritable bowel syndrome quality of life questionnaire; IBS-SSS, irritable bowel syndrome severity scoring system questionnaire; VAS, visual analogue scale; WMC, wireless motility capsule.

Supplementary Figure 2

The gas-sensing capsule system with a schematic of capsule internals: (**A**) Overview of the gas-sensing capsule system; (**B**) schematic of the gas-sensing capsule internals; (**C**) a typical readout.

Supplementary Figure 3

CONSORT participant flow diagram, adapted from So et al.[1]

Supplementary Figure 4

Overview of the number of capsule investigations of the wireless motility capsule and gas-sensing capsule: (**A**) flow diagram of the number of participants undertaking the investigations and the flow of capsules administered; (**B**) the number of successful capsule investigations undertaken across the dietary interventions. Two iterations of the gas-sensing capsule (Generations 1 and 2) were used. The Generation 1 gas-sensing capsule was introduced shortly following trial commencement, after active participants had already completed their first dietary intervention. The Generation 2 gas-sensing capsule with more advanced capabilities was introduced soon thereafter and used for the remainder of the trial. The estimation of hydrogen concentrations was technically unsatisfactory for the Generation 1 capsules and were excluded from analyses.

Supplementary Table 1

Mean daily nutrition profile of the dietary interventions, adapted from So et al.[1]

|  | **Control** | **Sugarcane** | **Combination** |
| --- | --- | --- | --- |
| Energy, MJ | 7.9 | 7.7 | 7.7 |
| Protein, g | 96.9 | 92.7 | 90.9 |
| Fat, g | 80.6 | 78.9 | 77.2 |
| Carbohydrate, g | 179.4 | 170.1 | 169.2 |
| Total fiber, g | 23.8 | 33.3 | 45.0 |
| Fiber, g | 22.1 | 31.1 | 32.4 |
| Resistant starch, g | 1.7 | 2.3 | 12.6 |
| Total FODMAPs, g | 1.84 | 1.66 | 1.55 |
| Fructans, g | 0.74 | 0.87 | 0.68 |
| Galacto-oligosaccharides, g | 0.48 | 0.71 | 0.57 |
| Polyols, g | 0.26 | 0.16 | 0.11 |
| Lactose, g | 0.67 | 0.67 | 0.67 |
| Fructose, in excess of glucose, g | 0.17 | 0.06 | 0.09 |

Supplementary References

1 So D, Yao CK, Ardalan ZS, Thwaites PA, Kalantar-Zadeh K, Gibson PR*, et al.* Supplementing Dietary Fibers With a Low FODMAP Diet in Irritable Bowel Syndrome: A Randomized Controlled Crossover Trial. Clin Gastroenterol Hepatol 2022;**20**:2112-20.e7.
